# Sedentary Behaviour in the Secure Forensic Hospital Setting: A Study from Dundrum Hospital Ireland

**DOI:** 10.1192/j.eurpsy.2022.1548

**Published:** 2022-09-01

**Authors:** A. Gibbons, T. Hoare, K. Kirrane, H. Kennedy, M. Davoren

**Affiliations:** 1 Dundrum Centre for Forensic Excellence, Central Mental Hospital And Trinity College Dublin, Dublin, Ireland; 2 Department of Psychiatry, Trinity College Dublin, Dublin, Ireland

**Keywords:** forensic psychiatry, Sedentary Behaviour, SIT-Q

## Abstract

**Introduction:**

Secure forensic mental health services offer care and treatment to mentally disordered offenders, with high rates of schizophrenia and major mental illness in these groups. Much of the excess morbidity and mortality seen among patients with schizophrenia is due to cardiovascular disease and obesity. Sedentary behaviour is associated with negative symptoms of schizophrenia and obesity.

**Objectives:**

The aim of this study was to ascertain the level of sedentary behaviour among inpatients in a secure forensic psychiatric hospital, Dundrum, Ireland, using a structured self-report measure of sedentary behaviours, the SIT-Q tool.

**Methods:**

A cross sectional study of self-reported sedentary behaviour was completed amongst the secure forensic inpatient population of Dundrum Hospital (N=94). Demographic details, details pertaining to diagnoses, ward level of dependency and length of stay were collated.

**Results:**

The majority of patients in the sample were male (89%) and the most common diagnosis was schizophrenia (71.7%). Mean age was 44.7 years (SD 11.42). 58.2% met criteria for obesity. We found high rates of self-reported sedentary behaviour across all wards of the service, with significantly high rates of sedentary behaviour being associated with screen time use in the hospital, including both personal screen time and therapeutic sessions based on screen time.

**Conclusions:**

Sedentary behaviour among in-patients in secure forensic hospitals is a significant issue. Measuring sedentary behaviour in a systematic manner is possible and identifies a potentially modifiable target to reduce co-morbidity and pre-mature mortality independent of other risk factors in this vulnerable patient group.

**Disclosure:**

No significant relationships.

